# Treatment Frequency and Dosing Interval of Ranibizumab and Aflibercept for Neovascular Age-Related Macular Degeneration in Routine Clinical Practice in the USA

**DOI:** 10.1371/journal.pone.0133968

**Published:** 2015-07-24

**Authors:** Alberto Ferreira, Alexandros Sagkriotis, Melvin Olson, Jingsong Lu, Charles Makin, Fran Milnes

**Affiliations:** 1 Novartis Pharma AG, Basel, Switzerland; 2 IMS Health, Plymouth Meeting, PA, United States of America; Tufts University, UNITED STATES

## Abstract

**Purpose:**

To compare treatment patterns of intravitreal ranibizumab and aflibercept for the management of neovascular age-related macular degeneration (nAMD) in a real-world setting over the first 12 months of treatment.

**Methods:**

A proprietary clinical database was used to identify treatment-naïve patients with nAMD in the USA with claims for ranibizumab or aflibercept between November 1, 2011 and November 30, 2013 and with follow-up of at least 12 months. Patients were considered treatment-naïve if they had no anti-VEGF treatment code for 6 months before the index date. Mean numbers of injections and of non-injection visits to a treating physician were compared between the two treatment cohorts (ranibizumab or aflibercept). In addition, the mean interval between doses was also investigated.

**Results:**

Patient characteristics were similar for those receiving either ranibizumab (n = 5421) or aflibercept (n = 3506) at the index date. The mean (± standard deviation) numbers of injections received by patients treated with ranibizumab (4.9 ± 3.3) or aflibercept (5.2 ± 2.9) were not clinically different. The mean number of non-injection visits was 2.8 ± 2.8 and 2.1 ± 2.5 for ranibizumab and aflibercept, respectively. Mean dosing interval was 51.0 days (± 41.8 days) in patients receiving ranibizumab and 54.1 days (± 36.0 days) in those receiving aflibercept. Results were robust to sensitivity analyses for definition of treatment-naïve, length of follow-up and treatment in the index eye only.

**Conclusions:**

Limited data exist regarding real-world treatment patterns of aflibercept for the management of nAMD. Our results suggest that, in routine clinical practice, patients receive a comparable number of injections in the first year of treatment with ranibizumab or aflibercept.

## Introduction

Age-related macular degeneration (AMD) is a chronic, degenerative eye disease that causes pathological changes in the macular region of the retina, leading to progressive loss of vision.[1] Neovascular AMD (nAMD), also known as wet or exudative AMD, accounts for 10–20% of all AMD cases, yet is responsible for 80−90%[2] of the severe vision loss associated with AMD.[3] This condition is characterized by abnormal growth and leakage of blood vessels in the macula, which causes central vision blurring and distortion (metamorphopsia).[4] nAMD is the leading cause of vision loss in people over 50 years old in industrialized nations, with a reported prevalence of 1−3% in elderly populations.[5,6,7] Without early diagnosis and treatment, nAMD can progress rapidly, leading to severe visual impairment and permanent vision loss in the affected eye. The economic burden associated with nAMD can therefore be considerable, reflecting both direct medical costs of diagnosis and treatment, and indirect costs associated with vision aids and care costs.[8,9]

Current standard care for the treatment of nAMD includes anti-vascular endothelial growth factor (anti-VEGF) therapies that inhibit the proliferation of blood vessels and reduce edema or swelling in the macula. Monthly dosing with ranibizumab, was shown to improve best corrected visual acuity (BCVA) by approximately seven and eleven letters on the Early Treatment Diabetic Retinopathy Study (ETDRS) chart at 12 months in the pivotal, phase 3 trials MARINA and ANCHOR, respectively, and improvements were generally sustained up to 24 months.[10,11,12] More recently, individualized dosing of ranibizumab has been shown to achieve similar gains in BCVA compared with monthly dosing,[13,14] and is recommended in most countries.[15] In the USA, treatment may be given either monthly or less frequently following administration of three monthly loading doses.[16]

Aflibercept has also been approved (by the US Food and Drug Administration in November 2011 and by the European Medicines Agency in November 2012) for treatment of nAMD, based on the results of the VIEW trials, which reported non-inferior efficacy for bimonthly aflibercept and monthly ranibizumab.[17] Aflibercept is recommended to be administered as three monthly loading doses, followed by intravitreal injection once every 8 weeks.[18,19]

It is unclear, in routine clinical practice, whether either drug is given as recommended. An analysis of US claims has recently reported that patients with nAMD received a mean of 6.1–6.9 ranibizumab injections in the first year of treatment,[20] which is lower than would be expected if patients were treated according to the label and comparable with that reported for individualized dosing in the recent Comparison of Age-related Macular Degeneration Treatments Trials (CATT) and HARBOR studies.[13,14] and in the 2013 Preferences and Trends (PAT) survey.[21] This claims database analysis of prescription records in the USA was performed to investigate prescribing practices for treatment-naïve patients with nAMD in routine clinical practice and to determine whether they differ for the currently approved treatments for nAMD, ranibizumab and aflibercept. At the time of this analysis, aflibercept had been available in routine clinical practice in the USA for 24 months, providing sufficient data for meaningful trends in treatment practice to be analyzed.

## Methods

This retrospective study was based on an analysis of US physician-level claims data from the Integrated Data Warehouse (IDW; managed by IMS Health, Pennsylvania‎, USA), a claims database that encompasses approximately 1 billion professional fee claims per year, representing approximately 80% of practicing eye care specialists (including over 13,000 ophthalmologists), and covering all 50 states. Of these physicians, approximately 25% met ‘physician stability’ criteria, defined as the consistent submission of medical claims to the IDW in the 6 months before index and throughout follow-up, and were included in the analysis. More than half of these stable physicians had prescribed ranibizumab and aflibercept during the study period. The data held in the database are obtained through agreements with electronic claims reprocessors and physician practices submitting claims through billing software. Approximately 95% of claims submitted for payment from these sources are available for analysis within 3 weeks. As an observational analysis of claims data, there was no ethic committee/institutional review board approval required for this study, nor patient consent required. All patient information held in the IDW is de-identified and Health Insurance Portability and Accountability Act of 1996-compliant; the investigators therefore had no access to personal patient information.

The study included adult patients with a first medical claim registered in the IDW with a procedure code for intravitreal injection of ranibizumab or aflibercept between November 1, 2011 and June 1, 2013, and with a concomitant diagnosis of nAMD (international classification of disease code: ICD-9-CM 362.52); this first claim was defined as the patient’s index date. Injections could be administered into the index eye and/or the fellow eye. Data were collected until November 30, 2013, to ensure at least 6 months of available follow-up data for all eligible patients. Patients were also required to have data recorded in the IDW for a minimum of 6 months before the index date (based on evidence of claims activity), and the physician administering the index medication needed to have consistently submitted medical claims to the IDW during the 6 months before the index date and during the follow-up period. Patients were excluded from the analysis if their records indicated that they had received more than one anti-VEGF drug on the index date (to avoid the potential confound of a patient being included in both groups); if they were younger than 18 years of age on the index date; or if the patient’s gender was not accurately identified on their records.

The primary analysis focused on treatment-naïve patients (defined as having received no anti-VEGF treatment claim in the 6 months before the index date) and who were treated continuously (i.e. received no other anti-VEGF therapy) with their index therapy for at least 12 months (365 days). In addition, instances of treatment with pegaptanib or bevacizumab were determined to identify: i) all patients who initiated first-line anti-VEGF therapy; and ii) patients who were switched to a different anti-VEGF therapy after initiation of ranibizumab or aflibercept therapy. The mean numbers of injections, non-injection visits and total number of visits to a treating physician during the first year after starting ranibizumab or aflibercept therapy were estimated. Negative binomial regression was used to compare the effect of patient characteristics on dosing interval estimates for those treated continuously with ranibizumab and aflibercept for at least 12 months (S1 Table).

**Fig 1:**
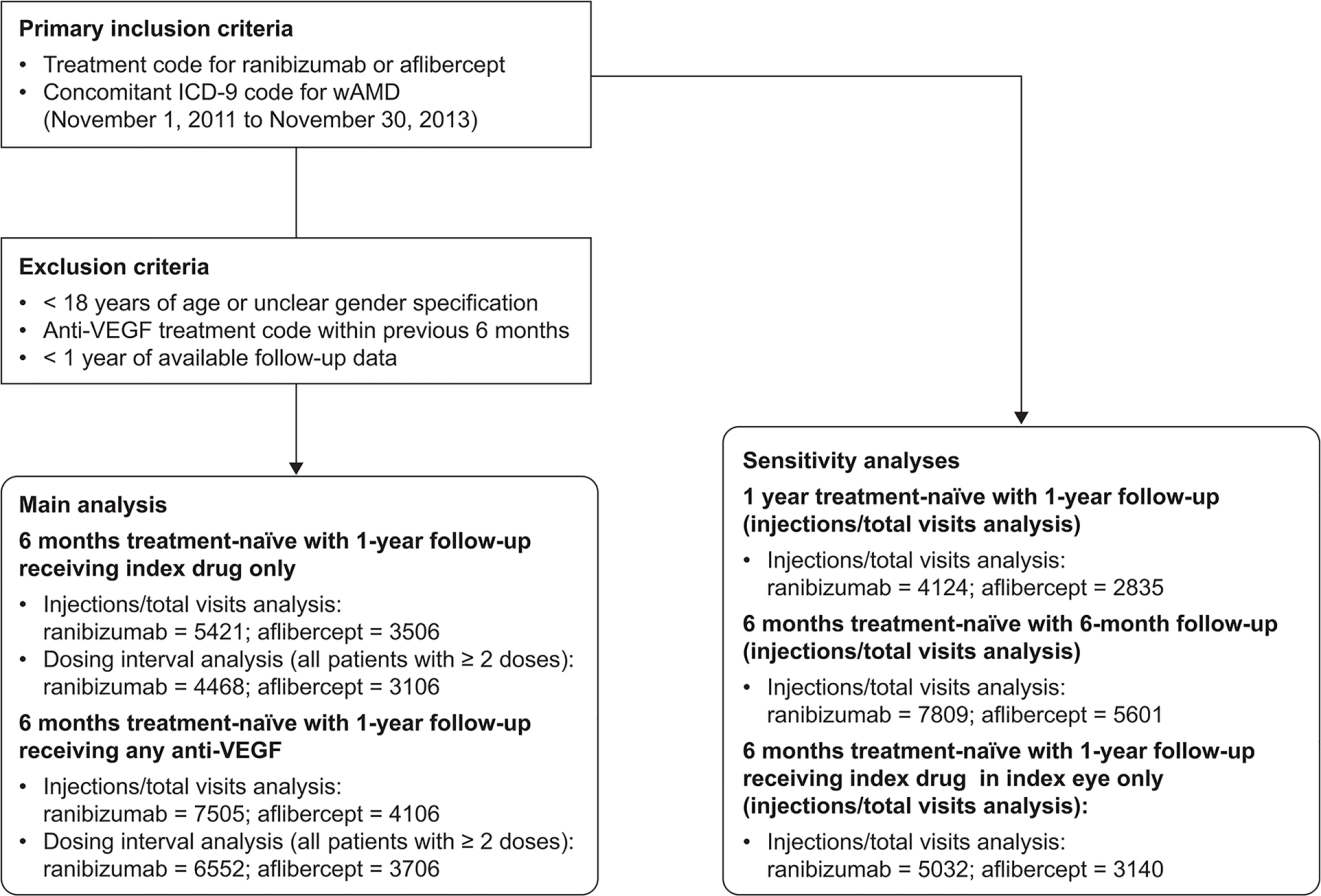
Evolution of Primary Analyses and Sensitivity Analyses Cohorts. ICD, international classification of disease code; nAMD, neovascular age-related macular degeneration; VEGF, vascular endothelial growth factor.

For the treatment interval analysis, all patients with at least two recorded doses were included (Fig 1). Mean dosing intervals (number of days between injections) were determined for the first year of therapy, for the first three doses (loading doses) and for doses 3−12 (maintenance doses) for patients starting on either treatment and receiving at least two injections. A generalized estimating equation (GEE) model was used to compare the effect of patient characteristics on dosing interval estimates for those treated continuously with ranibizumab and aflibercept for at least 12 months (S2 Table). Sensitivity analyses were performed: 1) using a longer definition of treatment-naïve (12 months instead of 6 months); 2) with a minimum follow-up of at least 6 months (instead of 12 months); and 3) including only injections received in the index eye. Between-group statistical differences were assessed using unpaired *t*-tests, with *p* < 0.05 used to define a significant difference and *p* < 0.0001 used to define a highly significant difference. Categorical variables were assessed using Χ-squared/Fisher’s exact test.

## Results

In total, 8927 patients were treated continuously with their index drug over 12 months (ranibizumab, n = 5421; aflibercept, n = 3506). The two treatment groups were comparable in terms of demographics or type of health plan, and almost all patients received treatment from an ophthalmologist (including retinal specialists) (Table 1). The majority of patients in both groups (ranibizumab, 65.0%; aflibercept, 62.9%) were female, and their mean ages were 79.5 years and 79.3 years, respectively. Cancer, chronic pulmonary disease and diabetes mellitus (without complications) were the only Charlson/Deyo comorbidities occurring in at least 5% of patients in either group. Claims were identified as being in the same eye (unilateral treatment) throughout follow-up in 92.8% (ranibizumab) and 89.6% (aflibercept) of patients.

**Table 1:** Patient Demographics and Baseline Characteristics Among Patients Receiving Ranibizumab or Aflibercept for the Management of nAMD.

	Ranibizumab (n = 5421)	Aflibercept (n = 3506)	*p* value
Proportion of patients in age group at index date, %			< 0.001^†^
< 65 years	4.2	3.2	
65–69 years	5.4	5.9	
70–74 years	9.7	12.1	
75–79 years	17.8	19.2	
80–84 years	25.5	25.2	
≥ 85 years	37.4	34.5	
Mean (SD) age, years	79.5 (7.2)	79.3 (6.7)	0.23
Proportion of female patients, %	65.0	62.9	0.04
Mean (SD) Charlson/Deyo Comorbidity Index	0.5 (1.0)	0.5 (1.0)	0.07
Incidence of Charlson/Deyo comorbidities in patients in sample cohort, %			
Diabetes mellitus (without complications)	9.3	8.6	0.26
Chronic pulmonary disease	7.1	7.1	0.91
Cancer	5.1	5.0	0.89
Cerebrovascular disease	4.4	4.3	0.83
Chronic heart failure	3.5	2.9	0.09
Renal disease	3.6	3.1	0.19
Peripheral vascular disease	2.6	2.7	0.84
Diabetes mellitus (with complications)	2.6	2.3	0.25
Rheumatologic disease	1.1	0.8	0.16
Dementia	0.4	0.3	0.77
Myocardial infarction	0.5	0.4	0.42
Peptic ulcer disease	0.3	0.3	0.86
Metastatic carcinoma	0.2	0.2	0.83
Mild liver disease	0.1	0.1	0.61
Specialty of physician prescribing index medication, %			
Ophthalmologist	99.0	98.4	0.02
Proportion of patients with health plan type, %			< 0.001^†^
Commercial	16.9	14.6	
Medicare	82.2	85.2	
Medicaid	0.9	0.2	
Proportion of patients from geographic region, %			< 0.0001^†^
Midwest	17.3	26.5	
Northeast	22.9	24.2	
South	48.0	42.3	
West	11.8	7.1	

nAMD, neovascular age-related macular degeneration; SD, standard deviation.

Between-group mean differences calculated by unpaired *t*-test; differences in categorical variables calculated by Χ-squared test/Fisher’s exact test.

^†^Comparison between treatment groups across categories.

**Fig 2:**
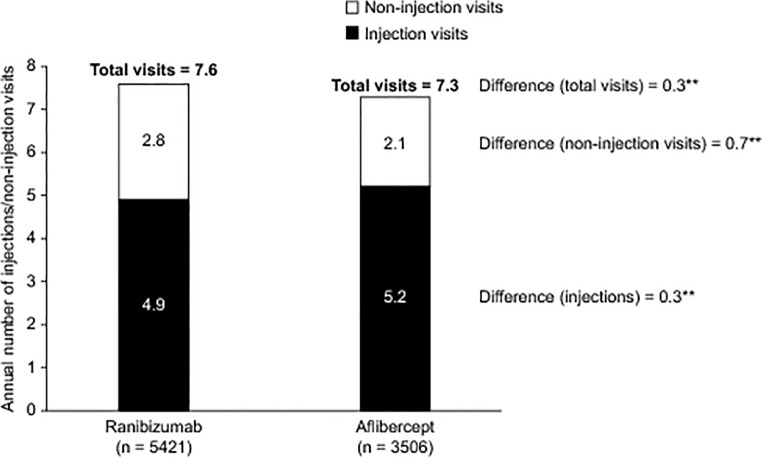
Annual Mean Number of Injections and Annual Mean Number of Non-injection Visits in the First Year of Therapy in Patients Receiving Treatment with Ranibizumab or Aflibercept for the Management of nAMD. Results are not adjusted for bilateral treatment. **p < 0.0001. nAMD, neovascular age-related macular degeneration.

**Fig 3:**
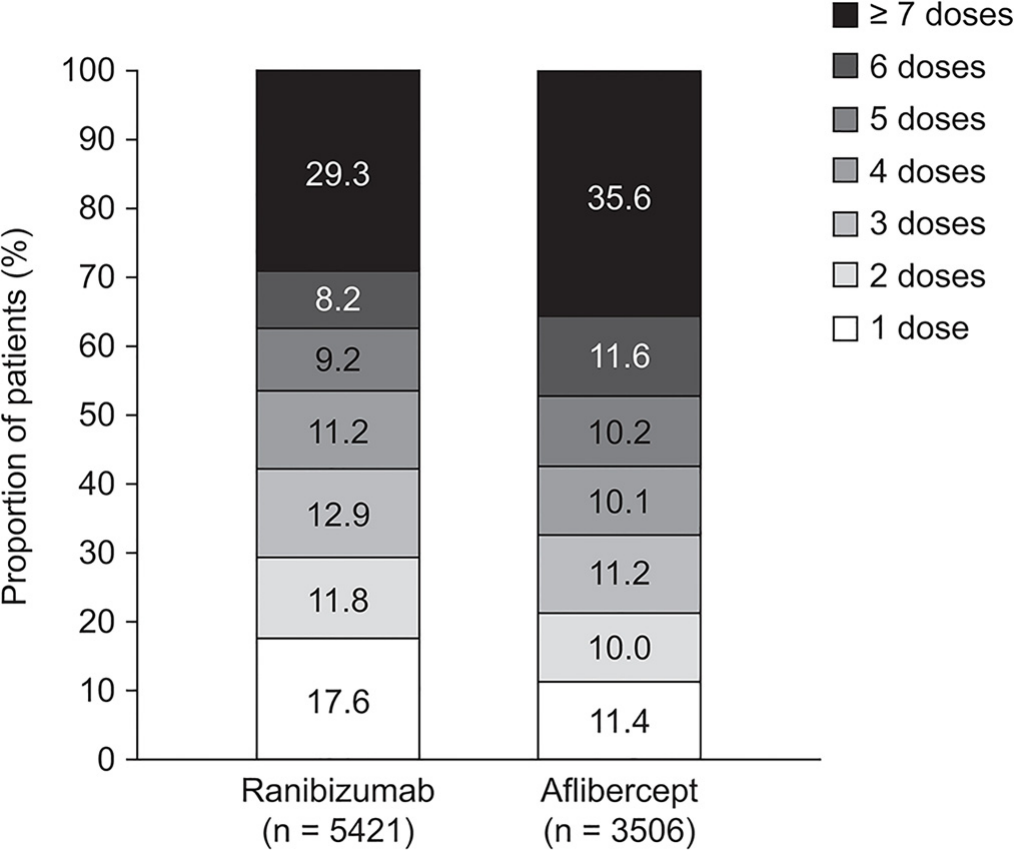
Dose Distribution of Index Medication Among Patients Receiving Ranibizumab and Aflibercept For Management of nAMD During 1-Year Follow-Up. nAMD, neovascular age-related macular degeneration.

For patients treated continuously with ranibizumab or aflibercept, the mean ± standard deviation number of injection visits during the first 12 months of treatment was 4.9 ± 3.3 and 5.2 ± 2.9, respectively (Fig 2). The mean number of non-injection visits was 2.8 ± 2.8 and 2.1 ± 2.5 for ranibizumab and aflibercept, respectively. The mean total number of visits to the treating physician (the sum of injection and non-injection visits) in the 12 months after the index date was 7.6 ± 3.7 and 7.3 ± 3.3, respectively. Over half of the patients in each group had four or more injections of their index drug within the first year of treatment (ranibizumab, 57.8%; aflibercept, 67.4%; Fig 3). The proportion of patients receiving ≤ 5 injections was 62.7% in the ranibizumab group compared with 53% of patients in the aflibercept group. Sensitivity analyses confirmed the results from the primary analyses (Tables 2 and 3).

**Table 2:** Number of Injections and Physician Visits Per Year.

Patient Cohort	N		Injection Visits (Per Year)		Total Visits (Per Year)	
	Ran	Afl	Ran	Afl	Ran	Afl
**Primary analyses**						
Patients treated continuously with index agent for ≥ 12 months of follow-up	5421	3506	4.9 ± 3.3	5.2 ± 2.9**	7.6 ± 3.7	7.3 ± 3.3**
Patients treated with any anti-VEGF agent for ≥ 12 months	7505	4106	5.6 ± 3.5	5.5 ± 3.1**	8.2 ± 3.8	7.7 ± 3.6**
**Sensitivity analyses**						
Patients treated continuously with index agent, ≥ 12-months treatment-naïve	4124	2835	5.0 ± 3.4	5.3 ± 3.0**	7.8 ± 3.9	7.4 ± 3.4**
Patients treated continuously with index agent for ≥ first 6 months of follow-up (annualized)	7809	5601	4.2 ± 3.0	4.7 ± 2.7**	6.9 ± 3.5	6.9 ± 3.2
Patients treated continuously with index agent in index eye only	5032	3140	4.8 ± 3.3	5.1 ± 2.9	7.6 ± 3.7	7.2 ± 3.3**

**p < 0.0001.

Afl, patients indexed to aflibercept; Ran, patients indexed to ranibizumab; VEGF, vascular endothelial growth factor.

In patients receiving more than one dose of their index treatment (ranibizumab, n = 4468; aflibercept, n = 3106), there was a difference of 3 days in the mean dosing intervals (51.0 ± 41.8 days for patients receiving ranibizumab and 54.1 ± 36.0 days for those receiving aflibercept) (Table 3). The proportion of patients who received all three loading doses of the index drug within the first 90 days was similar in the two treatment groups (aflibercept: 46.2%, ranibizumab: 43.1%; p = 0.51). The mean dosing interval between the first three doses was 54.5 ± 51.2 days for patients receiving ranibizumab and 52.0 ± 43.1 days for those receiving aflibercept (*p* < 0.01). Among the subset of patients who received four or more doses of the index drug (ranibizumab, n = 3132; aflibercept, n = 2363), 65.6% of ranibizumab initiators received all three loading doses during the first 90 days compared with 64.3% for aflibercept initiators. In addition, the mean dosing interval for maintenance doses was shorter for patients receiving ranibizumab (48.6 ± 34.0 days) than for those receiving aflibercept (55.4 ± 30.5 days) (*p* < 0.0001). Adjusting the primary analysis for baseline covariates did not affect the differences observed between treatment groups (S1 and S2 Tables).

**Table 3:** Dosing Intervals Between Intravitreal Injections.

Patient Group	N		Dosing Interval (Days Between Doses)		Mean Loading Dosing Interval (Days Between Doses)		Mean Maintenance Dosing Interval (Days Between Doses)	
			Ran	Afl	Ran	Afl	Ran	Afl
Patients treated continuously with index agent for ≥ first12 months of follow-up	4468	3106	51.0 ± 41.8	54.1 ± 36.0**	54.5 ± 51.2	52.0 ± 43.1*	48.6 ± 34.0	55.4 ± 30.5**
							(n = 3132)	(n = 2363)
Patients treated with any anti-VEGF agent for ≥ 12 months	6552	3706	49.2 ± 40.4	52.7 ± 36.9**	53.6 ± 50.5	52.9 ± 44.9	46.8 ± 33.1	52.5 ± 31.1**
							(n = 5006)	(n = 2887)

Afl, patients indexed to aflibercept; Ran, patients indexed to ranibizumab; VEGF, vascular endothelial growth factor.

Mean maintenance dosing interval includes only patients receiving ≥ 4 doses.

*p < 0.05

**p < 0.0001.

**Fig 4:**
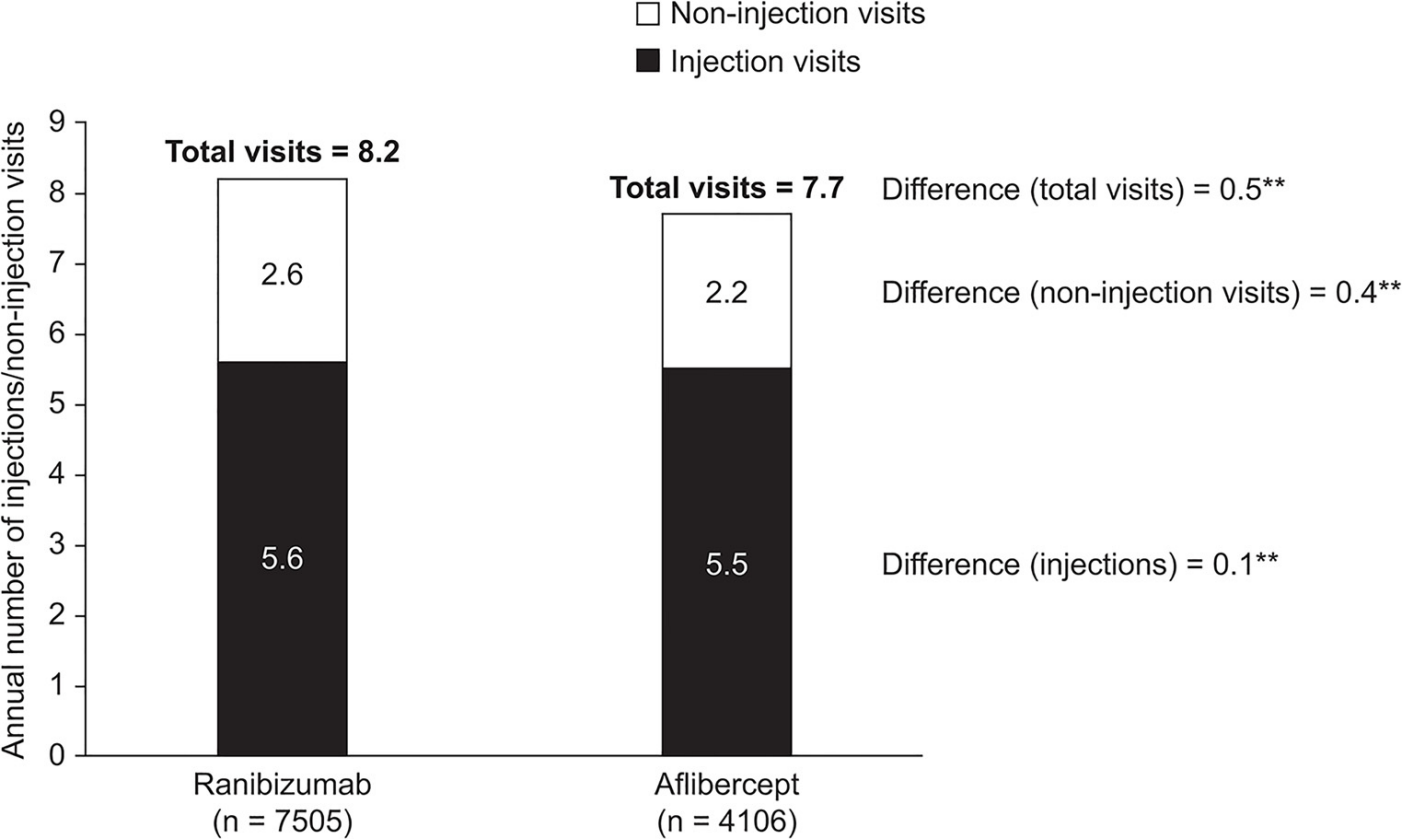
Annual Mean Number of Injections With any Anti-VEGF Treatment and Annual Mean Number of Non-injection Visits in The First Year of Therapy in Patients Starting Treatment with Ranibizumab or Aflibercept for Management of nAMD. Results are not adjusted for bilateral treatment and include patients who may have changed to another anti-VEGF treatment during the 1-year follow-up period. **p < 0.0001. nAMD, neovascular age-related macular degeneration; VEGF, vascular endothelial growth factor.

An additional analysis assessing all post-index injections of any anti-VEGF treatment in the 12 months after the index therapy was started (ranibizumab, n = 7505; aflibercept, n = 4106) found similar mean numbers of injections in the first 12 months of treatment regardless of index therapy (ranibizumab, 5.6 ± 3.5; aflibercept, 5.5 ± 3.1; Fig 4).

## Discussion

This database analysis of US physician-level claims data compared treatment patterns of ranibizumab and aflibercept in clinical practice. Results from this study suggest that ranibizumab and aflibercept are administered in a similar manner in the USA, despite different posology labels. For treatment-naïve patients treated continuously with ranibizumab or aflibercept for at least 12 months, there were no clinically meaningful differences in the mean number of injections or the total number of visits to a treating physician.

Aflibercept is recommended to be given as three monthly loading doses followed by bimonthly treatment, resulting in a total of seven injections per year for the first year of treatment.[18,19] In contrast, according to our study, patients received a mean of 5.2 injections annually if treated continuously with aflibercept. In the USA, ranibizumab is recommended to be administered as monthly intravitreal injections or less frequent than monthly after three loading doses with regular assessment.[16] Additionally, in recent clinical trials using individualized dosing, patients received a mean of 6.9[14] or 7.7[13] injections in the first year of treatment. These values are higher than the 4.9 injections observed annually in our study for patients treated continuously with ranibizumab. However, the mean number of injections reported for ranibizumab in our study concurs with that reported in other observational studies. A study of ranibizumab prescription performed in the USA reported that patients received a mean of 6.1–6.9 ranibizumab injections in the first year of treatment.[20] Several prospective observational studies across Europe have reported results supportive of the present findings, with patients with nAMD receiving a mean of 4.3–6.0 ranibizumab injections annually in Belgian, German, Dutch, Swedish and UK databases.[22,23,24] These findings are further supported by a recent analysis assessing treatment patterns in a different US claims database, which reported that 5.8 (ranibizumab) and 5.5 (aflibercept) injections were given annually, although the patient cohort was substantially smaller (ranibizumab n = 347; aflibercept n = 57) than in our study.[25]

When assessing patients who had changed anti-VEGF therapy after their index injection, the number of any anti-VEGF injections received after index were comparable, whether patients started on ranibizumab or aflibercept treatment. The numbers of injections and of total visits were also comparable to those observed in patients treated continuously with their index agent during follow-up. These results suggest that patients who changed therapy did not begin their new treatment with a loading period, because the number of injections received was comparable to that for patients treated with a single agent. Further studies are needed to understand whether this applies to all patients or whether this reflects differential behavior of individual physicians.

The difference in mean dosing intervals between ranibizumab and aflibercept over the first 12 months of treatment was only 3 days (51.0 days vs 54.1 days, respectively). The mean dosing interval for loading doses was 54.5 days for ranibizumab and 52.0 days for aflibercept, indicating that for many patients receiving either treatment, the interval between loading doses is greater than the 1-month interval recommended for both treatments. After loading doses, the mean dosing intervals for aflibercept (55.4 days) and ranibizumab (48.6 days) were lower than the bimonthly (56 days) dosing recommended for aflibercept. This suggests that some patients required aflibercept more frequently than recommended on the label. It should be noted that the mean frequency of treatment in this study (aflibercept, 5.2 injections; ranibizumab, 4.9 injections) is comparable to that in year 2 of the VIEW studies (aflibercept, 4.2 injections; ranibizumab, 4.7 injections).[26]

This study is, to our knowledge, the largest database study to compare treatment patterns of ranibizumab and aflibercept in routine clinical practice. Aflibercept has been available for use in clinical practice in the USA since November 2011. To perform a meaningful assessment of treatment patterns for aflibercept, we chose a data source that would provide up-to-date prescription data for aflibercept from its launch in the USA. The IDW was chosen because it provides rapid processing of claims data: approximately 95% of claims submitted for payment are available for analysis within 3 weeks of the service date. This ensured the availability of sufficient data on aflibercept use. It is important to consider that the statistically significant differences reported in this study are most likely a consequence of the large number of patients included and do not necessarily reflect clinically relevant differences. Additional strengths of this study include the extensive sensitivity analyses, which found that changes in the definition of treatment-naïve, the follow-up period and whether results were restricted to injections in the index eye only made no appreciable difference to the results or to the interpretation of findings.

The study has a number of limitations. First, the analyses did not adjust for bilateral treatment. Sensitivity analyses were performed looking at the index eye and showed that the results were robust and consistent. Second, the study used physician-level computerized coded data to assess treatment patterns; as such, a small risk of misclassification is inevitable, although we believe that any such misclassification applies to both treatment groups equally. Selection bias is a potential issue with any database study, and in light of reports that aflibercept works well as “salvage” therapy” in patients who are resistant to ranibizumab or bevacizumab therapy,[27] it can be argued that a different mindset may exist for aflibercept use. Third, the treatment patterns observed in the present study may not necessarily represent those in other countries or in other indications. Further studies of treatment patterns in other countries and indications are thus warranted when sufficient data for aflibercept become available. Finally, claims databases do not include data on outcomes such as visual acuity or anatomic features, pigment epithelial detachment or hemorrhage; thus, additional data sources are needed to correlate injection frequency with visual outcomes. These analyses are beyond the scope of this type of study, and require further investigation.

In conclusion, the present study is, to our knowledge, the largest database analysis comparing treatment patterns of ranibizumab and aflibercept, two key treatments in combatting the loss of vision associated with nAMD. These data suggest that, to date, ophthalmologists in the USA generally administer both treatments at intervals that differ from those recommended on the label. In real-world clinical practice, physicians tend to follow treatment regimens that are different from those suggested by randomized controlled trials, such as “as needed” (pro re nata [PRN]) or treat and extend (T&E). This is of particular interest given the different length of experience physicians are likely to have with each agent. Given that current market prices for ranibizumab and aflibercept are similar and that injections constitute the majority of the treatment costs associated with these treatments,[28] the observation that the number of injections administered for each treatment is broadly comparable suggests that budgetary considerations for both treatments are likely to be similar in routine clinical practice. However, this will need to be confirmed by cost-effectiveness analyses of visual outcomes of nAMD patients treated with ranibizumab and aflibercept. Further studies are warranted to compare treatment outcomes and treatment patterns for both agents in routine clinical practice as more data become available.

## Supporting Information

S1 Table Negative Binomial Regression Adjustments for Injections/Number of Visits: Relative Rates for Baseline Demographic Variables. Results shown are relative rates for injection and visit values for the primary analysis cohort. *p < 0.05; **p < 0.0001.
(DOCX)

S2 Table Generalized Estimating Equation Model Adjustment for Mean Dosing Intervals: Relative Rates for Baseline Demographic Variables. Results shown are relative rates for mean dosing intervals for the primary analysis cohort. *p < 0.05; **p < 0.0001.
(DOCX)
