# Correction: Treatment Frequency and Dosing Interval of Ranibizumab and Aflibercept for Neovascular Age-Related Macular Degeneration in Routine Clinical Practice in the USA

**DOI:** 10.1371/journal.pone.0136515

**Published:** 2015-08-21

**Authors:** The PLOS ONE Staff

## Notice of Republication

This article was republished on August 7, 2015, to replace Fig 2, which was incorrect. The publisher apologizes for the error. Please download this article again to view the correct version.
