# Supplementary material for: Treatment Frequency and Dosing Interval of Ranibizumab and Aflibercept for Neovascular Age-Related Macular Degeneration in Routine Clinical Practice in the USA
Source: PLoS One. 2015 Jul 24;10(7):e0133968. doi: 10.1371/journal.pone.0133968 (PMC4514835; doi:10.1371/journal.pone.0133968)
Supplement: S1 Table — Results shown are relative rates for injection and visit values for the primary analysis cohort. *p < 0.05; **p < 0.0001. (DOCX) [file pone.0133968.s001.docx]

## Supplementary Table S1. Negative Binomial Regression Adjustments for Injections/Number of Visits: Relative Rates for Baseline Demographic Variables.

|  | **Relative Rate** | | |
| --- | --- | --- | --- |
| **Independent Variables** | **Injection Visits** | **Total Visits** | **Non-injection Visits** |
| **Study cohort comparisons, aflibercept**  **compared with ranibizumab** | | | |
| Aflibercept (treatment-naïve) | 1.08** | 0.96** | 0.75** |
| Age, years |  |  |  |
| < 65 | 0.86* | 1.01 | 1.29* |
| 65–69 | 1.01 | 1.07* | 1.16* |
| 70–74 | 1.06* | 1.05* | 1.02 |
| 75–79 | 1.06* | 1.05* | 1.03 |
| 80–84 | 1.03 | 1.01 | 0.96 |
| > 85 (reference category) | 1 | 1 | 1 |
| Gender |  |  |  |
| Female | 1.02 | 1.02 | 1.00 |
| Male (reference category) | 1 | 1 | 1 |
| Charlson/Deyo Comorbidity Index | 0.97 | 0.99 | 1.03 |
| Health plan type |  |  |  |
| Medicaid | 1.63** | 1.43** | 1.15 |
| Medicare | 1.01 | 1.00 | 1.00 |
| Commercial (reference category) | 1 | 1 | 1 |
| Geographic region |  |  |  |
| Midwest | 0.96* | 1.06** | 1.24** |
| Northeast | 0.95* | 0.95* | 0.94 |
| West | 1.00 | 1.03 | 1.09* |
| South (reference category) | 1 | 1 | 1 |

Results shown are relative rates for injection and visit values for the primary analysis cohort.

**p* < 0.05; ***p* < 0.0001.
