# Supplementary material for: Treatment Frequency and Dosing Interval of Ranibizumab and Aflibercept for Neovascular Age-Related Macular Degeneration in Routine Clinical Practice in the USA
Source: PLoS One. 2015 Jul 24;10(7):e0133968. doi: 10.1371/journal.pone.0133968 (PMC4514835; doi:10.1371/journal.pone.0133968)
Supplement: S2 Table — Results shown are relative rates for mean dosing intervals for the primary analysis cohort. *p < 0.05; **p < 0.0001. (DOCX) [file pone.0133968.s002.docx]

## Supplementary Table S2. Generalized Estimating Equation Model Adjustment for Mean Dosing Intervals: Relative Rates for Baseline Demographic Variables.

| **Independent Variables** | **Mean Dosing Interval Relative Rate** | |
| --- | --- | --- |
| **Study cohort comparisons, aflibercept**  **compared with ranibizumab** | | |
| Aflibercept (treatment-naïve) | | 1.10** |
| Age, years | | |
| < 65 | | 0.92 |
| 65–69 | | 0.24* |
| 70–74 | | 0.97 |
| 75–79 | | 0.95* |
| 80–84 | | 0.99 |
| > 85 (reference category) | | 1 |
| Gender | | |
| Female | | 0.93* |
| Male (reference category) | | 1 |
| Charlson/Deyo Comorbidity Index | | 1.03** |
| Health plan type | | |
| Medicaid | | 0.75* |
| Medicare | | 0.96 |
| Commercial (reference category) | | 1 |
| Geographic region | | |
| Midwest | | 1.10* |
| Northeast | | 1.14** |
| West | | 1.14** |
| South (reference category) | | 1 |

Results shown are relative rates for mean dosing intervals for the primary analysis cohort.

**p* < 0.05; ***p* < 0.0001.
